# Optical trapping of mesoscale particles and atoms in hollow-core optical fibers: principle and applications

**DOI:** 10.1038/s41377-025-01801-5

**Published:** 2025-03-31

**Authors:** Rui Wang, Wei Li, Zhiwen Xia, Hongchang Deng, Yao Zhang, Rongxin Fu, Shuailong Zhang, Tijmen G. Euser, Libo Yuan, Ningfang Song, Yi Jiang, Shangran Xie

**Affiliations:** 1 Key Laboratory of Photonic Information Technology (Ministry of Industry and Information Technology), School of Optics and Photonics, Beijing Institute of Technology, Beijing, China; 2 School of Instrumentation and Optoelectronic Engineering, Beihang University, Beijing, China; 3 Photonics Research Center, Guilin University of Electronics Technology, Guilin, China; 4 Engineering Research Center of Integrated Acousto-opto-electronic Microsystems (Ministry of Education of China), School of Integrated Circuits and Electronics, Beijing Institute of Technology, Beijing, China; 5 Nanophotonics Centre, Department of Physics, Cavendish Laboratory, University of Cambridge, Cambridge, UK

**Keywords:** Optical manipulation and tweezers, Fibre optics and optical communications

## Abstract

Hollow-core fiber (HCF) is a special optical waveguide type that can guide light in the air or liquid core surrounded by properly designed cladding structures. The guiding modes of the fiber can generate sufficient optical gradient forces to balance the gravity of the particles or confine the atom clouds, forming a stable optical trap in the hollow core. The levitated objects can be propelled over the fiber length along the beam axis through an imbalance of the optical scattering forces or by forming an optical lattice by the counter-propagating beams. The ability to overcome the diffraction of the laser beam in HCF can significantly increase the range of the optical manipulation compared with standard free-space optical tweezers, opening up vast ranges of applications that require long-distance optical control. Since the first demonstration of optical trapping in HCF, hollow-core-fiber-based optical trap (HCF-OT) has become an essential branch of optical tweezer that draws intense research interests. Fast progress on the fundamental principle and applied aspects of HCF-OT has been visible over the past two decades. In recent years, significant milestones in reducing the propagation loss of HCF have been achieved, making HCF an attractive topic in the field of optics and photonics. This further promotes the research and applications of HCF-OT. This review starts from the mechanism of light guidance of HCF, mainly focusing on the issues related to the optical trap in the hollow core. The basic principles and key features of HCF-OT, from optical levitation to manipulation and the detection of macroscopic particles and atoms, are summarized in detail. The key applications of HCF-OT, the challenges and future directions of the technique are also discussed.

## Introduction

Optical tweezers are one of the most fast-growing areas in optics and photonics^1–3^. The features of high manipulation precision^4^, multi-dimensional motion control^5–9^, and the capability of integrating the setup with highly sensitive optical measurement techniques in both spatial and spectral domains^10,11^ have made the optical tweezer an indispensable tool for exploring new phenomena at the micro- and nano-scales^12^. Conventional single-beam optical tweezers, first demonstrated by Arthur Ashkin in 1986, use a highly focused laser beam to provide sufficient optical gradient forces to balance the gravity and the axial scattering forces experienced by the dielectric particle^13,14^. Since then, the form of optical tweezers has been extended to a vast range of configurations, including dual-beam traps^15,16^, multi-beam traps^17,18^, near-field traps^19–24^, holographic optical traps^25–27^, fiber-based optical trap^28–31^, etc. The different configures of the optical tweezers have led to a wide range of applications in fundamental physics^32–39^, precision sensing^40–43^, biological particle manipulation and analysis^44–48^, colloid and interface science^49^, and particle characterization^11,50,51^. From the fundamental aspects, the ability to manipulate both the translational and rotational motions of the trapped objects via regulation of the trapping beam intensity, profile, and angular momentum has led to dramatic processes in the field of levitomechanics^52^. Cavity or feedback cooling approaches have been applied to suppress the thermal motion of the particle in a vacuum environment^53–56^, promoting the demonstration of ground-state cooling of the center-of-mass motion of the levitated nanoparticles^57–59^. The creation of multiple trapping potentials can be exploited to investigate the tunable coupling dynamics of the bound particles^60,61^.

Hollow-core fiber (HCF) is an emerging waveguide platform for investigating light-matter interaction. Even though capillary fibers have been used in optics and photonics, the realm of HCF was largely empowered by the invention of hollow-core photonic crystal fiber (HC-PCF) by Philip Russell at the University of Bath^62^, permitting the sufficient guidance of light in the hollow core. Since then, HCF has been widely used in the field of ultrafast lasers^63,64^, gas photonics^65–67^, optical communication^68^, quantum optics^69,70^, and fiber sensing^71–78^. The hollow core can be filled with gases or liquids to widely tune the parameters of light-matter interaction. More importantly, light can be tightly confined over the entire length of the fiber, overcoming the diffraction of the laser beam and resulting in an infinite focusing length. This property significantly increases the interaction length and, thus, the strength of the interaction in HCFs. This has led to the generation of intense and broadband laser sources ranging from deep-UV to mid-IR^79^.

**Fig. 1:**
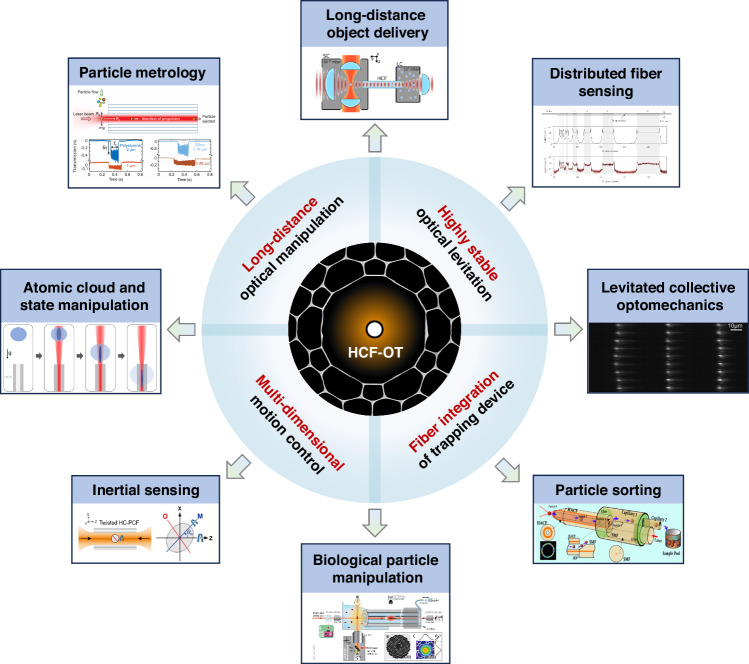
Features and applications of HCF-OT technique^6,71,82,83,95,162,163^

Under the context of the optical tweezers, the guiding modes of the HCF, including both the fundamental and the higher-order core modes (HOMs), can be used to levitate the objects (particles or atoms) in the hollow core close to the core center. In this situation, the objects can be optically guided along the fiber over a long distance, significantly increasing the range of optical manipulation^80,81^. This feature makes hollow-core-fiber-based optical trap (HCF-OT) very different from conventional optical tweezers, unlocking several unique application scenarios. It can be used to realize a long-range object delivery to places difficult to access by conventional optical tweezers^82^. An array of dielectric particles can be trapped in HCF with the inter-particle distances tunable from micrometer to millimeter range^83,84^, offering unique possibility to investigate long-range optical binding dynamics and multi-parameter sensing. HCF-OT can also form a remote sensor to overcome the limitation of the conventional distributed fiber sensors^71^. The confinement of the trapping field in the hollow core can introduce a self-induced back-action mechanism to enhance the optical trapping forces and, thus, the trapping stability of the objects^85^. Since its first demonstration in capillary fiber^86^ and later in HC-PCF^87^, HCF-OT has been extensively studied by many groups worldwide^86–95^, the fundamental and application aspects have been greatly extended over the past two decades (Fig. 1).

This article intends to provide a substantial review of the state-of-the-art HCF-OT technique, aiming to provide an overview and summary of its properties, principles, and progress of the method. Section 2 explains the guidance principle of HCFs, including the photonic bandgap fiber (HC-PBGF) and anti-resonant guidance fiber (HC-ARF), which has drawn more intense interest in recent years. Section 3 describes the fundamental principles of trapping mesoscale particles and atoms in the hollow core, and the key features of the HCF-OT technique are summarized. Section 4 discusses several key applications of HCF-OT, and Section 5 lists future directions and challenges of the technique.

## Principle of light guidance in HCF

Capillary fiber is the simplest form of the HCF in which light can be guided in the air core by reflection at the air-silica boundary under grazing incidence. A small amount of light hence can be transmitted in the glass region of the capillary during reflection, resulting in light attenuation along the propagation^96^. The fundamental mode of the core has a Bessel shape. Therefore, a particle in the bore is attracted by the high intensity of the core mode on the capillary axis and is propelled in the propagation direction of light. Due to its simplicity, a capillary with a 20 μm inner diameter was the first hollow waveguide used for optical manipulation^86^. In this initial experiment, atoms were guided by red-detuned light in the evacuated bore of a capillary over 3 cm. Capillary fiber was also used for the light guidance of mesoscale solid particles and liquid droplets over 2 cm^88^. The relatively high loss of the capillary fiber (>100 dB/m for core diameter of ~30 μm^79^) significantly limits the propulsion length of the objects.

**Fig. 2:**
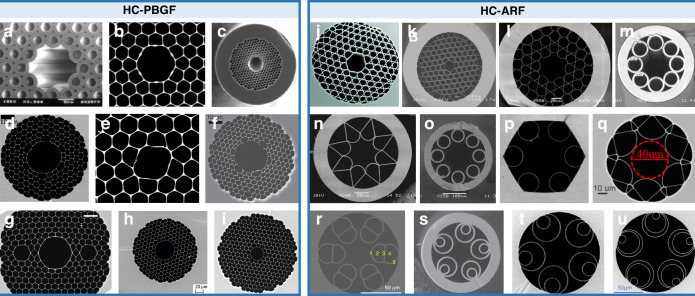
Typical hollow-core photonic bandgap fiber (HC-PBGF, left panel) and anti-resonant fibers (HC-ARF, right panel). **a** First reported HCF^62^ (reprinted with permission from AAAS). **b** Low-loss 7-cell fiber^180^ (reproduced with permission from Springer Nature). **c** Low-loss 19-cell fiber^181^ (reprinted with permission from © Optica Publishing Group). **d** Low-loss five-ring HCF^182^ (reprinted with permission from © Optica Publishing Group). **e** Highly birefringent 4-cell fiber^183^. **f** Surface-mode free 19-cell fiber^184^ (reproduced with permission from Springer Nature). **g** Polarization maintaining HCF^185^ (reproduced with permission from Springer Nature). **h** Low-loss 19-cell fiber for mid-IR operation^186^ (reprinted with permission from © Optica Publishing Group). **i** Commercially available 7-cell fiber^187^. **j** Kagomé-style HCF^65^ (reprinted with permission from AAAS). **k** Large-pitch single-cell-core Kagomé fiber^67^ (reprinted with permission from AAAS). **l** Hypocycloid-core Kagomé fiber^188^ (reprinted with permission from © Optica Publishing Group). **m** Negative curvature fiber^189^ (reprinted with permission from © Optica Publishing Group). **n** Negative curvature fiber^190^. **o** Revolver HCF^103^. **p** Effectively single-mode six-tube fiber^191^ (reprinted with permission from © Optica Publishing Group). **q** Lotus fiber^192^ (reprinted with permission from © Optica Publishing Group). **r** Conjoined-tube fiber^193^ (reproduced with permission from Springer Nature). **s** Nested fiber^194^ (reprinted with permission from Turpion). **t** Low-loss (0.22 dB/km) 5-tube HC-ARF^195^ (reprinted with permission from F. Poletti). **u** Record low-loss double nested HC-ARF^196^ (reprinted with permission from F. Poletti)

To reduce the guiding loss of the fiber, cladding structures are introduced (thus forming HC-PCF) to provide better confinement on the light in the core. The principle of HC-PCF guidance can be categorized into photonic bandgap and anti-resonant guidance^97,98^. In a photonic bandgap structure, a periodic arrangement of cladding refractive index on the wavelength scale is introduced such that light within a specific wavevector range cannot propagate through those photonic crystals. Laser light within the wavelength range is forbidden to enter the cladding and thus can be tightly confined in the core. Some examples of cross-sectional images of PBGFs reported in the literature are shown in Fig. 2(a–f). There are excellent reviews into how HC-PBGFs guide light and their properties^99,100^. In terms of optical trapping, HC-PBGF can confine light within a relatively small core, offering a higher beam intensity and, thus, stronger optical forces. Relatively small trapping power is required to balance the particle gravity. Another benefit of the HC-PBGF is the relatively small bending loss of the fiber, making it more convenient to handle especially in situations involving long-distance manipulation^98^. On the other hand, the relatively narrow guidance window of HC-PBGF makes it less convenient to launch other wavelengths for particle detection or spectrum measurement for particle characterization.

**Fig. 3:**
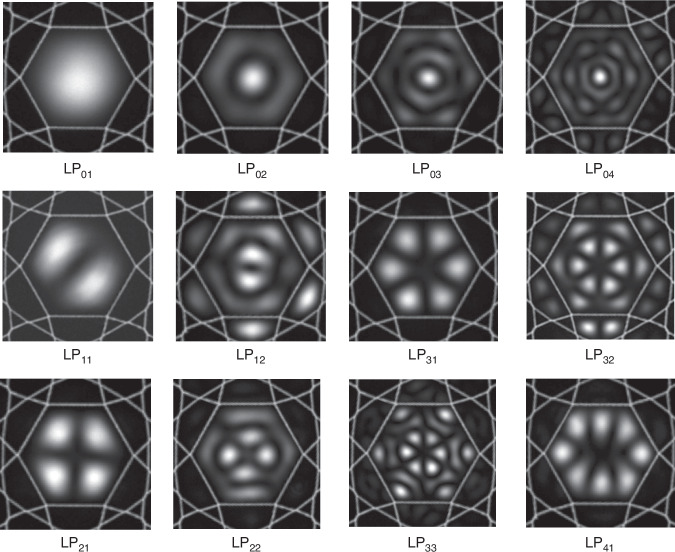
Excited HOMs in HC-ARFs through a prim-coupling technique from the side of the fiber^111^

Unlike HC-PBGF, anti-resonant fiber (HC-ARF) relies on the antiresonances generated from the thin glass membranes making up the cladding^65,67^. Initial Kagomé-style HCFs have many layers of air channels in the cladding^65^. It is later discovered that the first layer close to the hollow core dominates the contribution of the mode guidance window and the addition of further rings of air holes to the structure does not seem to reduce the loss of the fibers^101,102^. This realization has led to many reports of fibers with effectively a single ring of air holes in the cladding but with relatively uncompromised loss performance^103,104^. The thin-wall capillaries around the core provide a series of resonant wavelengths in analogy to the Fabry–Pérot interferometers^67^:

1 $${\lambda }_{l}=\frac{2t}{l}\sqrt{{n}^{2}-1},l=1,2,3,\cdot \cdot \cdot \cdot \cdot \cdot$$

where *t* and *n* are the thickness and refractive index of the capillary wall, and *l* is the integer number representing the order of the resonances. Laser light at the resonant wavelength can be strongly coupled to the capillaries and introduce high loss on the fiber transmission. At an anti-resonant wavelength, the reflectivity is optimal, resulting in a low-loss transmission window of the fiber. By introducing nested tubes to enhance light confinement in the hollow core, the loss of the HC-ARF has been significantly improved^105–107^, approaching a level even lower than that of the standard telecommunication fibers in the C-band^108^. The low-loss guidance of HC-ARF is very suitable for ultra-long distance (over meters long) particle guidance. The broadband, often octave-spanning operational bandwidths of HC-ARF, make it ideal to transmit another wavelength (in addition to the trapping wavelength) to probe the trapped particles’ properties and motion. Compared with the HC-PBGF, the HC-ARF’s straightforward structure also introduces less distortion when performing optical imaging of the particle from the side of the fiber, which could be beneficial for particle metrology. HOMs can also be guided in the hollow core^109,110^, adding new degrees of freedom for tailoring the optical forces. Figure 3 displays the measured HOMs in the Kagomé-style HCF, excited by a prim-coupling technique from the side of the fiber^111^. The multiple lobes of the HOMs in the fiber cross-section could act as tighter trapping sites for particles due to the higher intensity of the smaller lobes, thus forming a two-dimensional particle array.

## Principle and key features of HCF-OT

**Fig. 4:**
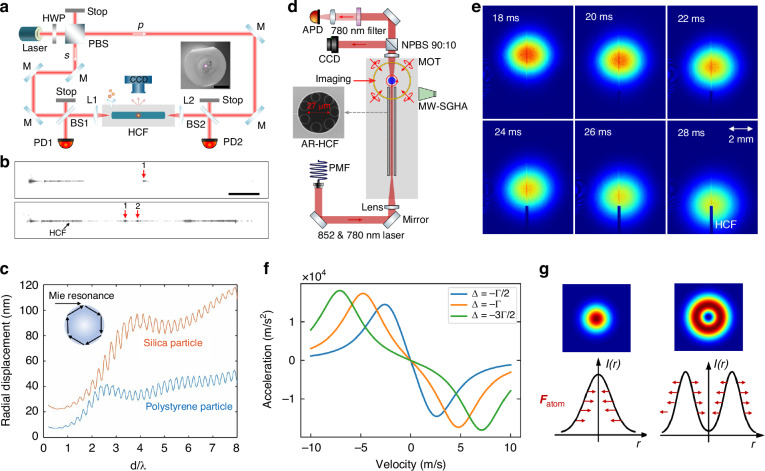
Principle of neutral particles and atom trapping in HCF. **a** Experimental setup of particle trapping in HCF^94^. **b** Optical image of single and two 2 μm-diameter polystyrene particles trapped in HCF^94^. Scale bar: 1 cm. **c** Simulated radial displacement of silica and polystyrene particles of different diameters trapped in a HCF with 20 μm core diameter and under 1 W of trapping power. **d** Experimental setup of atom trapping in HCF^93^. **e** The absorption images of atomic clouds captured by CCD at different dropping times^93^. **f** The acceleration of moving rubidium atoms in counter-propagating near-resonance laser fields caused by the scattering forces. **g** FORT based on a red-detuned Gaussian beam and a blue-detuned dark hollow beam

HCF-OT commonly uses a dual-beam optical trap to achieve the manipulation of mesoscale particles or atoms over the fiber (see Fig. 4a, b). For the neutral dielectric particles, the optical gradient forces are accounted to balance the particle gravity given the mode profiles in the hollow core. In the Rayleigh regime in which the particle diameter is much smaller than the trapping wavelength, the optical gradient force ***F***_grad_ can be calculated using the dipole approximation^13^:

2 $${{\boldsymbol{F}}}_{grad}(r)=\frac{1}{4}\frac{{\alpha }_{d}}{c{\varepsilon }_{0}}\nabla {I}_{i}(r)$$

where *α*_d_ is the real part of the complex particle polarizability, *c* is the vacuum speed of light, *ε*_0_ is the dielectric permittivity of the vacuum, and *I*_i_ is the intensity of the trapping beam, which is a function of the radial coordinate *r* over the cross-section. The intensity profile of the fundamental mode of HCF can be expressed as a Bessel function that has the highest intensity in the core center^96^. In the Mie regime in which the particle diameter is comparable to the trapping wavelength, even though empirical formula to estimate the optical force exists^112^, the explicit optical forces need to be calculated using numerical approaches, such as *T*-matrix^113^, discrete dipole approximation^114^, and finite element modeling^115^. It is revealed that arbitrary electric fields can be expressed in terms of the superposition of the core modes (instead of general spherical harmonic functions), which can significantly facilitate the calculation of the optical forces in the Mie regime in HCF^116^. Figure 4c plots the simulated radial displacement of the silica and polystyrene particles of different diameters trapped in a HCF with 20 μm core diameter and under 1 W of trapping power. It can be seen that in both Rayleigh and Mie regimes, the range of particle displacement is almost within 0.1% of core radius, indicating that the particle can be well considered as being levitated in the core center. The ripples observed for particles with *d*/*λ* > 1 are induced by the Mie resonances within the particles^117,118^. For larger particle (i.e. *d*/*λ* > 1), under the geometric optical approximation, constructive interferences can occur at specific diameter if the light circulating in the sphere due to internal reflection add in phase (see inset of Fig. 4c). When the particle diameter is smaller (i.e. *d*/*λ* < 1), the radiation loss of the Mie modes increases in the wave optic model, leading to a significant drop on the quality factor of the resonance and thus the disappearing of the interference patterns.

The optical scattering forces generated from the counter-propagating beams are balanced to achieve particle propulsion over the fiber. Two possible configurations can be used to realize the particle manipulation. When the counter-propagating beams’ polarization is parallel, a standing wave is formed along the fiber, and the particles can be trapped at positions of maximum intensity. In this case, the function of particle transportation can be achieved by tuning the phase difference between the counter-propagating beams through a phase modulator. When the states of polarization of the counter-propagating beam are set orthogonal to each other through, for instance, by using a polarization beam splitter, particle propulsion can be achieved by the imbalance of optical power of the two beams and, thus, the corresponding scattering forces. In this case, an optical conveyor belt based on multimode beating effect can be realized^119^, which can be used to control the particle motion with an additional degree of freedom.

In the case of optically trapping of atoms, the force on a two-level atom moving quasi-classically in a monochromatic laser beam can be determined as^120^:

3 $${\boldsymbol{F}}=\hslash {\boldsymbol{k}}\frac{\Gamma }{2}\frac{s}{1+s+4{\Delta }^{2}/{\Gamma }^{2}}-\frac{\hslash \Delta }{2}\frac{\nabla s}{1+s+4{\Delta }^{2}/{\Gamma }^{2}}$$

where Γ is the natural linewidth of the atomic energy level, *s* = *I*(*r*) / *I*_s_ is the saturation parameter, *I*(*r*) is the intensity of the laser beam, *I*_s_ = *πhc*Γ/(3*λ*^3^) is the saturation intensity, $\hslash$ is the reduced Planck constant, and Δ = *ω* − *ω*_0_ is the detuning of the driving field frequency *ω* from the atomic resonance frequency *ω*_0_. The first term on the right-hand side of Eq. (3) is denoted as the radiation force, originating from the scattering of photons by the atom. The second term is the dipole force or gradient force, which arises from the intensity gradient of the optical field.

The experimental configuration of atom trapping is sketched in Fig. 4d. By turning on the trapping laser, atoms with temperature lower than the dipole potential are trapped and guided into the HCF under gravity. The absorption images of atomic clouds are captured by CCD at different dropping times (Fig. 4e). For atoms moving in a far-off resonance light field, the dipole force is the dominant force, the magnitude of which is determined by the intensity gradient of the optical field. As shown in Fig. 4f, when the driving laser field frequency is red-detuned (Δ < 0), the potential energy is negative, and the dipole force on the atom points in the direction of increasing light intensity. Conversely, when the driving laser field frequency is blue-detuned (Δ>0), the dipole trap potential energy is positive, and the dipole force points in the direction of decreasing light intensity. By controlling the frequency detuning and the intensity gradient of the driving optical field, one can achieve a red-detuned optical dipole trap using a Gaussian beam or a blue-detuned optical dipole trap using a dark hollow beam (Fig. 4g). Increasing the detuning effectively reduces the photon scattering rate for a given trap depth. Therefore, in applying optical dipole traps, a far-off resonance trap (FORT) is commonly used to reduce spontaneous emission rates and avoid atomic decoherence caused by photon scattering.

For both mesoscale particles and atom clouds, the HCF-OT technique has several unique advantages over its free-space counterparts, which can be outlined as follows:

**Long-distance optical manipulation**. The guidance of both optical mode and objects along the fiber opens up the possibility of manipulating the particle over the entire length of the fiber. The particle manipulation range can reach meters or even longer, which can be used for targeted object delivery and distributed sensing. The platform can also be scaled up to trap a long chain of tens or even hundreds of particles within the same HCF to investigate collective binding dynamics of the particle array.

**Highly stable optical levitation**. The hollow core acts as a sufficient shield for environmental airflow and perturbations increasing the trapping stability. This especially helps to keep the particle in the trap once the environmental pressure is varied (e.g. during the vacuum pumping process). In addition, due to the field confinement given by the core wall, the motion of the particles in the fiber cross-section could strongly modulate the optical field distribution in the core, which acts as a back action and can increase the optical gradient forces by orders of magnitude. The effect has been demonstrated by trapping a mechanically compliant nanospike inside the hollow core^85^.

**Multidimensional control of particle motion**. The excitation of both the fundamental and HOMs can be used to precisely tailor both the amplitude and the directions of the optical gradient and scattering forces experienced by the particles^116,121^. The optical torque applied to the particles can also be manipulated through the controlled excitation of the HOMs with distinct amplitude, phase and polarization distributions over the fiber cross section^6^. The complex radial mode distributions, which may vary over a short distance in the case of free space optics, can be maintained along the fiber through a sophisticated fiber design^122^.

**Fully integration of the trapping setup**. The possibility of integrating the trap inside the hollow core enables a highly compact version of the optical tweezer. The in-coupling and out-coupling optics can be arranged in a fiber pigtail, and the fiber, in principle, can be sealed to form a compact device^66,123^. The potential of a high degree of integration may unlock some applications sensitive to dimension and the overall mass of the device.

### Optical manipulation of micro- and nano-particles in HCF

#### Particle loading process

To achieve robust optical manipulation of mesoscale particles in HCF, the first key issue is to efficiently load particles from free space into the hollow core, a process in which the capture dynamics of the particles in front of the HCF end face play an important role. One sufficient and frequently used approach in experiments follows the aerosol launching procedure^124^. The particles are first dissolved in water in a particle-to-water mass ratio of 10^-3^ to 10^-2^. A medical nebulizer produces droplets of the particle solution, each containing a single particle on average. The droplets are injected into the chamber via an inlet tube in the lid placed above the front face of the fiber until one is trapped in front of the hollow core. Laser irradiation usually evaporates the droplet within a few seconds, leaving the particle in the dual-beam optical trap. A trapping event can be observed either through a drop in transmitted power (from fractions of a per cent for 100 nm particle up to 5% for 1 µm particle) or by observing changes in the image of the fiber end face taken by an external camera. This aerosol launching approach typically applies to particles of <5 μm diameter, determined by the mesh of the nebulizer. Bigger particles can be launched by using a glass plate mounted on a piezoelectric transducer, which is placed below the fiber end face^71^. The piezoelectric transducer can be driven at the resonance frequency of the glass plate, catapulting the particles in front of the fiber.

**Fig. 5:**
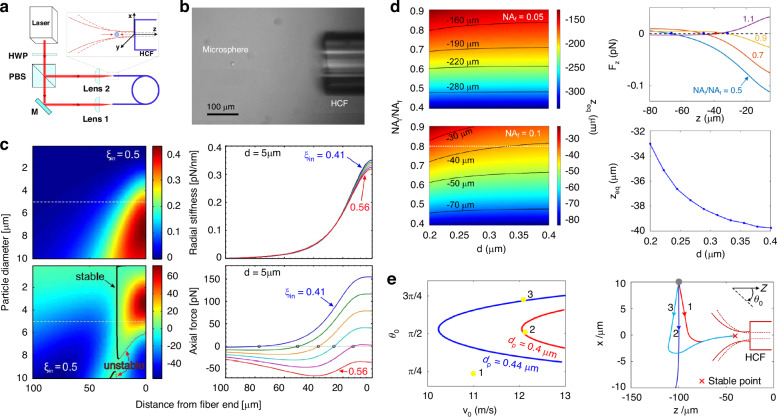
Optical trapping and capture process of dielectric particles in front of the HCF end face. **a** Sketch of the experimental configuration. **b** Optical image of a 15-μm-diameter polystyrene particle trapped at the HCF end face^126^. **c** Plots of axial trap stiffness and optical force versus distance from the fiber end under different particle diameters and relative power of the counter-propagating trapping beams^125^. **d** Contour lines of the distance from the fiber end of the equilibrium trapping point versus particle diameter and NA_l_/NA_f_. **e** Conditions of the initial amplitude and direction of particle launch velocity for successfully capturing silica particles with different diameters and the corresponding trajectories of the particle capture process

Once the particle is released, it may be stably trapped in front of the fiber end face. The optical trap formed in front of the HCF end face involves a diverging beam exiting from the HCF (dashed red line in the inset of Fig. 5a) and a converging beam coupled into the hollow core from free space (solid red line). In such a configuration, the condition of forming a stable trap and the trapping position depends on the particle diameter and the trapping power. Schmidt et al. calculated the results of the radial optical trap stiffness *k*_z_ and the axial optical force *F*_z_ under different particle diameters and the relative power of the counter-propagating trapping beams (*ξ*_in_ = *P*_in_/(*P*_in_+ *P*_out_), where *P*_in_ and *P*_out_ are the power of the inward and outward beams)^125^. The net axial force for varying particle diameter and distance from the fiber is depicted. The contour curves in the bottom-left figure of Fig. 5c indicate positions where the counteracting axial forces balance for equal optical powers. Those equilibrium positions can be stable (solid black curve) or unstable (dotted red curve). Under the simulation condition (hollow core diameter *D* = 12 μm, total trapping power of 1 W), The stable trapping position for microspheres up to 8 μm in diameter is almost independent of particle size and located 26 μm in front of the fiber end facet, where the beam diameters and thus the intensities of the counter-propagating beams are equal. The axial trapping position can be tuned by varying *ξ*_in_ as shown by the encircled zero-crossings in the bottom-right panel of Fig. 5c. Increasing the power of the in-coupled beam pushes the *d* = 5 μm particle towards the fiber end face until it is finally launched for *ξ*_in_ > 0.54. There is no stable capture point when *ξ*_in_ > 0.54. The axial stiffness (slope at zero-crossing) is two orders of magnitude lower than the radial stiffness, resulting in an asymmetric three-dimensional trapping force landscape that is elongated in axial direction.

In addition, the axial trapping condition and position are also sensitive to the numerical aperture of HCF (NA_f_) and the coupling lens (NA_l_)^94^. It can be seen from Fig. 5(d) that when NA_l_/NA_f_ is less than unity, stable trapping points are present, and the equilibrium position becomes closer to the fiber end face when NA_l_/NA_f_ is approaching unity. In contrast, when NA_l_/NA_f_ is >1, the slope *∂F*_*z*_/*∂z* is positive when *F*_z_ = 0, indicating that it is an unstable trapping position. Thus, when the counterpropagating beams have equal power, it is necessary to satisfy NA_l_ < NA_f_ to achieve stable trapping in front of the HCF endface. After the particle is trapped in three dimensions, it can be reliably pushed into and out of the hollow core by changing the power ratio of the counter-propagating beams. Figure 5b shows the typical optical image of the polystyrene particles with a diameter of 15 µm stably trapped in front of the fiber end face^126^.

The dynamic capture process of the particle in front of the HCF endface can be precisely modeled by solving the equations of motion in the *x*-*z* plane (see Fig. 5a):

4 $$\begin{array}{c}{m}_{p}\frac{{d}^{2}x}{d{t}^{2}}+3\pi \eta {d}_{p}\frac{dx}{dt}={F}_{grad,x}-{m}_{p}g\\ {m}_{p}\frac{{d}^{2}z}{d{t}^{2}}+3\pi \eta {d}_{p}\frac{dz}{dt}={F}_{grad,z}+{F}_{scat,z}\end{array}$$

where *m*_p_ and *d*_p_ are the mass and hydrodynamic diameter of the particle, *η* is the viscosity of air, *g* is gravitational acceleration, *F*_grad,x_ and *F*_grad,z_ is the optical gradient forces in *x* and *z* directions, *F*_scat,z_ is the optical scattering forces in the z direction. The Runge–Kutta method can be applied for numerically solving Eq. (4), from which the particle trajectory during the capture process can be explicitly obtained. The right panel of Fig. 5e displays the calculated trajectories of the loaded particles under different incidence angles and initial velocities. The model can be used to predict feasible parameter ranges of the initial amplitude and direction of particle launch velocity based on trajectory analysis to achieve successful particle capture. The analysis can provide guidance for improving the particle-loading efficiencies of the HCF-OT.

#### Tracking of the particle motion

Once the particle is trapped in the hollow core, its motion can be monitored by measuring the scattered light from the fiber side or endface. The two-dimensional transverse motion of the particle across the fiber cross-section can be monitored by setting a quadrant photodetector at the fiber endface to measure the center-of-mass displacement of the output beam from the core caused by the oscillating particle^71^. It is also possible to measure the radial displacement from the side of the fiber by using a CCD camera or a quadrant photodetector. In this case, the particle motion in the imaging plane of the detector can be directly read from the detector, and the motion in the axial direction is expected to vary the imaging clarity or the collected intensity by adding a pinhole in front of a photodetector.

**Fig. 6:**
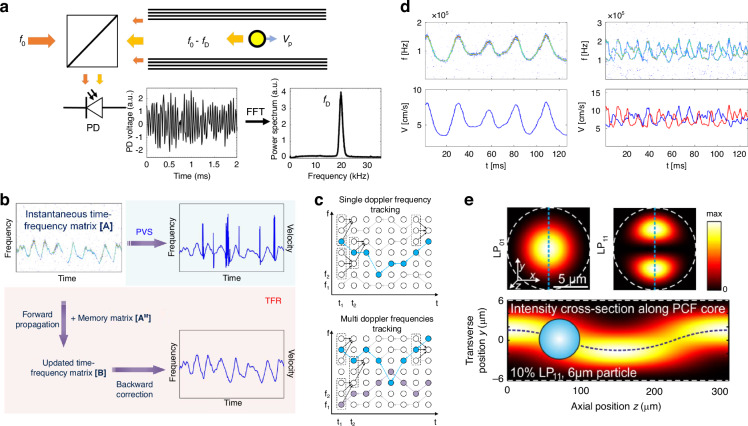
Doppler velocimetry of the propelled particles in HCF. **a** Principle of Doppler velocimetry of optically propelled particle in HCF. **b** Comparison of conventional peak-value searching (PVS) and the time-frequency ridge (TFR) methods to extract the Doppler frequencies^94^. **c** Sketch of the TFR algorithm for tracking single and multiple Doppler frequency peaks^94^. **d** The measured Doppler frequencies and the corresponding axial velocities of single and two polystyrene particles with 2 μm diameter^94^. **e** Top: calculated intensity patterns of the fundamental and higher order modes. Bottom: intensity distribution of mode mixture^119^

The axial motion of the particle can be tracked using Doppler velocimetry^125,127^. For a non-relativistic particle moving with axial velocity *v*_z_, the frequency of its backscattered light is shifted by *f*_D_ with respect to the incident beam:

5 $${f}_{D}=\frac{2}{\lambda }{v}_{z}$$

where *λ* is the trapping wavelength. Thus, the Doppler frequency shift is proportional to the particle axial velocity. As sketched in Fig. 6a, *f*_D_ can be obtained by measuring the beat signal between the backscattered light from the moving particle and the fiber end face reflection. The time domain signal collected by the photodiode is transformed into the frequency domain by a short-time Fourier transform operation, from which the Doppler frequency shift can be retrieved. Conventional peak-value searching method suffers from significant errors when the signal-to-noise ratio (SNR) of the Doppler spectrum is not sufficient for a perfect Lorentzian fit. To solve this issue, Wang et al. proposed a non-Markovian Doppler velocimetry, which applies a time-frequency ridge algorithm to extract the instantaneous frequency^94^. The lower panel of Fig. 6b sketches the procedure of the TFR algorithm, including a forward-propagation and a backward-correction process. In the forward-propagation process, an updated time-frequency matrix is generated, the elements of which are the weighted summation of elements in the original time-frequency matrix and the Doppler spectrum in the previous time moments introducing the memory effect (Fig. 6c, d). The memory effect can improve the accuracy of particle velocity tracking by more than two orders of magnitude in the low SNR regime and can resolve the issue of multiple-particle velocity extraction^94^. The position of the particle along the fiber can be further obtained through temporal integration of the measured instantaneous axial velocity using the Doppler velocimetry^71^. The periodic variation of the particle instantaneous axial velocity (see Fig. 6d) is induced by the modal beating effect in HCF. As shown in Fig. 6e, a suitably chosen coherent superposition of co-propagating LP_01_ and LP_11_ modes, together with an uncorrelated backward-propagating LP_01_ mode, results in a stiffness-tunable three-dimensional optical trap^119^. The field intensity on the particle, and thus the axial component of the optical force, peaks at the off-center positions, resulting in a speed variation with a period of *L*_B_/2, where *L*_B_ is the intermodal beat length between LP_01_ and LP_11_ modes.

### Atom trapping in HCFs

**Fig. 7:**
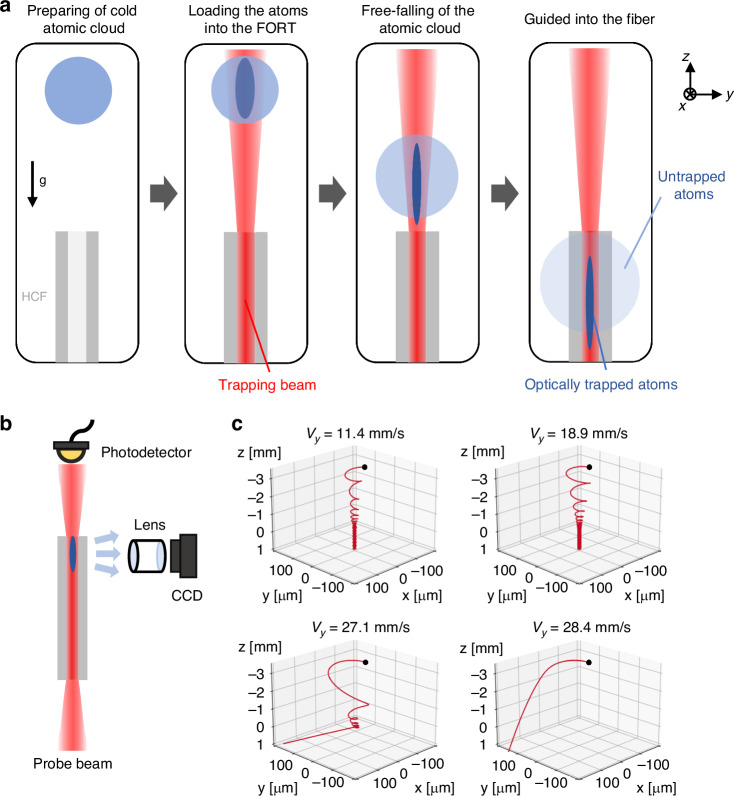
Loading and detection of atoms in HCF. **a** Typical procedures of loading cold atoms into HCF. **b** Two types of detection method for trapped atoms inside HCF. **c** Typical motion trajectories of atoms moving in FORT with different initial velocity conditions. *v*_x_ and *v*_z_ were set zero in the simulation. The black dots mark the initial positions of the atoms

For experiments of loading cold atoms into HCF, it is typically necessary to use a relatively high-power Gaussian beam, which causes a large potential depth to enhance the loading efficiency. Atoms can be considered to move in an axisymmetric circular potential trap. Depending on the initial velocity conditions, the motion of atoms can be classified into three types of trajectories: quasi-linear trajectories, quasi-circular trajectories, and elliptical precession trajectories. Figure 7c illustrates the typical motion trajectories of atoms moving in a FORT with different initial velocities. In the simulation, *v*_x_ and *v*_z_ were set zero and *v*_y_ was increased. As the initial velocity of the atoms gradually increases, the atoms begin to “escape” from the dipole trap created by the trapping beam. Typically, the loading efficiency is in the range of 0.1% – 3%, depending mainly on the geometric overlapping between the atomic cloud and the trapping beam^128–130^.

#### Loading of cold atoms into HCF

Typical procedures of loading cold atoms into HCF are shown in Fig. 7a. Atoms are first laser-cooled in a magneto-optical trap (MOT) with a final temperature of several micro-Kelvin, and an atomic cloud with a diameter of several millimeters is trapped in front of the HCF end face. Then, the MOT is turned off, and a far-off-resonance trapping beam is adiabatically turned on, forming a funnel-like dipole trap. The atoms are loaded into the FORT by superimposing the atomic cloud with the trap volume. The optically trapped atoms are guided towards the core of the HCF due to both the dipole force and gravity. The radial temperature of the trapped atoms is usually a fraction of the potential depth of the dipole trap. Once guided inside the core, the atoms move freely in the axial direction while remaining confined in the radial direction, preventing collision with the inner core wall.

#### Detection of optically trapped atoms inside HCF

Figure 7b shows that the detection of optically trapped atoms inside HCF can be implemented from both axial and radial directions^129–131^. A probe beam near resonance is sent through the fiber in the axial direction, and the transmission spectrum is monitored. This can accurately estimate the number of atoms inside the fiber and the radial temperature of the atomic ensemble. Radial detection is based on fluorescence imaging, which can be used to determine the location of atoms during transportation.

As for axial detection, two probe laser pulses will be successively sent into the HCF. The first pulse detects the absorption by the atoms, and the second pulse serves as a reference. A photodetector with high responsivity and low noise, such as an avalanche photodiode (APD) or photomultiplier (PMT), is placed at the other end of the fiber to detect the probe pulses. By scanning the frequency of the probe laser, the transmission spectrum can be measured, and the resonant optical depth is obtained^130^. The radial temperature of the atomic ensemble and the number of atoms can be estimated with time-of-flight measurement by releasing the cloud for some time before measuring the transmission spectrum. Radial detection uses a resonant probe beam to excite the atoms. The scattered photons transmitted through the side of the fiber are recorded with a CCD camera. The centroid position of the atoms can be resolved according to the fluorescence images^131^.

## Applications of HCF-OT

The unique features of HCF-OT, as listed in Section 3, have led to several special and primary applications that may be difficult to access with conventional optical tweezers.

### Long-distance object delivery

**Fig. 8:**
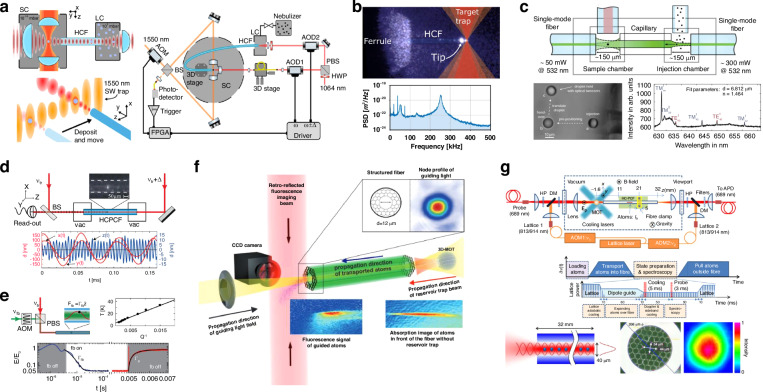
Long-distance propulsion of particles and atoms in HCF. **a** Schematic diagram of HCF loading device of nanoparticles into ultra-high vacuum^82^. **b** Video results of particle transmission and the corresponding power spectral density of the signal detected^82^. **c** Sketch of the aerosol trap and glycerol-water droplet handover results and CERS spectrum of a glycerol-water droplet held in optical tweezers^123^ (reprinted with permission from Royal Society of Chemistry). **d** Experimental setup: An HCF connects two vacuum chambers (vac)^89^. **e** Feedback cooling of the nanoparticle center-of-mass motion inside HCF^89^. **f** Transport of cold atoms over HCF^132^. **g** Controlled transport of cold atoms into HCF using a moving optical lattice^133^

Through optical forces, HCF-OT can deliver micro- and nano-objects along the fiber. Grass et al. demonstrated long-range control and feedback cooling of particle motion under the condition of pressure gradient in HCF. The detection of the three-dimensional motion of trapped nanoparticles in HCF was realized by the measuring the transmitted light, and the cooling were achieved by launching in an additional beam (Fig. 8d, e)^89^. This technique has been further used to load the nanoparticles into the vacuum environment. Lindner et al. demonstrated the direct loading of particles with a diameter of 143 nm to 365 nm into an ultra-high vacuum chamber using the standing wave optical trap formed in HCF. The accurate position of nanoparticle was controlled by an acousto-optic deflector over the optical conveyor belt (Fig. 8a, b)^82^. From the application aspect, particle guidance through a capillary fiber has been used to deliver single aerosols from an injection chamber to a separated measurement chamber. Horstmann et al. proposed a fiber capillary trap that can transport particles with a diameter of 500 nm to 19 μm to a designated position^91^. The proposed chip integration can conveniently deliver the particle to another fiber-integrated cavity-enhanced Raman spectrum setup, in which standard optical fiber was used to transmit the pump laser and to collect the Raman signal, as shown in Fig. 8c^123^.

In addition, the atom clouds can also be transported over HCF. Vorrath et al. demonstrated the first guiding of cold atoms through an 88 mm-long HC-PBGF^132^. An 8.2 mK trapping potential within a 12 μm core diameter HC-PBGF is obtained using a 1067 nm far-red-detuned guiding beam. A peak atomic flux of 10^5^ atoms/s is observed, and a constant atomic flux of 1.5 × 10^4^ atoms/s for over 150 ms is maintained by introducing an extra optical dipole trap at the fiber tip. The result demonstrates the feasibility of long-distance transport of cold atoms over HCF. Cold atoms can also be transported into HCF in a controlled manner using a moving optical lattice^133^. As illustrated in Fig. 8g, two 813 nm lattice beams with a frequency difference of δ*v*(t) are coupled into both ends of a Kagomé-style HCF, creating a potential well depth of approximately 30 μK at MOT and about 300 μK inside the fiber. Approximately 104 atoms, initially trapped in a MOT, were loaded into the optical lattice and transported into the fiber by tuning the frequency difference of the lattice beams.

### Collective optomechanics of levitated particle array

**Fig. 9:**
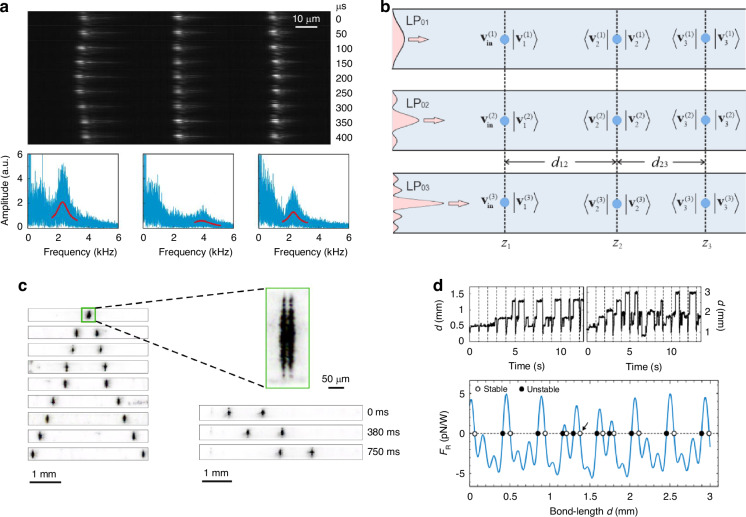
Optical manipulation of an array of dielectric particles in HCF. **a** A series of snapshots of the bound-particle array during one period of the breathing mode, captured with a high-speed camera. The lower panel plots the measured spectra of the mechanical motion of the three bound particles. The blue curves are the measured data, and the red curves are Lorentzian fits^83^. **b** Sketch of the intermodal scattering and beating process^83^. **c** Side-view of configurations with bond lengths between 42 μm and 3 mm^84^. **d** Changes in bond length when perturbed with a train of control pulses with peak power 10 mW (left) and 40 mW (right). The lower panel plots the calculated repulsive optical force (*F*_R_) between the particles under 1 W of total trapping power as a function of bond length. Stable spacings are marked with open circles, and unstable ones with black dots^84^

The ability to trap multiple particles in the hollow core offers a unique platform for investigating the rich optomechanical dynamics of arrays of levitated particles in a well-isolated and protected environment. Multiple trapping sites have previously been created using interference^134^ and holographic tweezers^135^ in free-space configuration, allowing the formation of a lattice of trapped particles. Optical binding is possible in a 1D particle array because the optical fields propagate bidirectionally along the array. Most 1D optical binding experiments have been performed in free space over distances limited by the Rayleigh range of the focusing optics. However, to control and measure the collective binding dynamics of such arrays, it is necessary to manipulate and monitor individual particles within the array without perturbing other degrees of freedom. This requires extended inter-particle distances and long-range interactions between trapped particles. In recent years, the optical binding range has been expanded using non-diffracting Bessel beams^136^ or by trapping particles in the evanescent field near the surface of a multimode glass microfiber^137^. In those experiments, external spatial light modulators were used to create binding sites, limiting the stability and power handling of the system. HCF-OT provides an ideal solution for resolving this issue. Bykov et al. have reported the observation of long-range optical binding of multiple microparticles inside the evacuated core of HC-PBG^83^. In this experiment, three polystyrene particles with 1 μm diameter are stably bound together with an inter-particle distance of ~40 μm, or 50 times longer than the wavelength of the trapping laser (Fig. 9(a)). The optical binding effect in HCF is mediated by intermodal scattering and interference, as illustrated in Fig. 9b. The levitated particle in the hollow core can be viewed as a mode converter that scatters the incident fundamental mode to the HOMs. The intermodal beating effect can further create additional trapping sites for the subsequent particles. The particles are bound to each other through optical forces, and the motion of each particle in three dimensions is expected to modulate the optical field and, thus, the forces acting on all particles. The bound-particle array can be translated to and fro over centimeter distances along the fiber. In addition, the collective mechanical modes of the bound-particle array could be observed when the hollow core was evacuated to 6 mbar gas pressure (Fig. 9a). The measured inter-particle distance at equilibrium and mechanical eigenfrequencies is supported by an analytical formalism modeling the dynamics of the binding process, demonstrating the capability of HCF-OT to investigate collective optomechanical dynamics. To further adjust the inter-particle distances and thus the optical binding forces between the particles, Sharma et al. have demonstrated the reconfigurable millimeter-range optical binding of polystyrene microparticles by using the group velocity walk-off effect of the core modes. The inter-particle distance can be varied from 40 μm to over 3 mm by unbalancing the powers in the counterpropagating trapping beams due to the use of weakly chirped pulses and HC-ARF with low group velocity dispersion (Fig. 9c, d)^84^.

### Atomic physics

The HCF-OT atom trapping techniques discussed in Section 3.2 have enabled several quantum and atomic physics applications. One of the earliest uses of HCF in atom optics experiments was the demonstration of electromagnetically induced transparency (EIT) in a thermal rubidium vapor in a HC-PBGF by Ghosh et al. in 2006^138^. The authors used a novel light-induced atomic desorption to release Rb atoms into the core. Due to the confined geometry, this resulted in a very high atom density, enabling EIT at a 1000 times lower threshold compared to bulk geometries. In 2011, Bajcsy et al. loaded ultracold rubidium vapor in a HC-PBGF. The atoms were laterally trapped by a red-detuned dipole trap, achieving a significantly enhanced optical depth of around 180, further reducing the power required for optical switching.

In 2014, Epple et al.^139^ embedded highly excited cesium Rydberg atoms in a Kagomé HCF. Rydberg atoms have at least one electron with a very high quantum number, resulting in an extremely large polarizability. This makes them of great interest for applications in quantum- computing, simulation, and metrology. Epple et al. demonstrated that Rydberg excitations can be achieved at room temperature within a HCF^139^. They noted that the diameter of the fiber core affects the Rydberg states due to the effects of stray fields from surface charges that detune the Rydberg excitation. In 2017, these surface effects were directly measured using higher-order spatial modes, excited by a spatial light modulator^140^. Veit et al. then demonstrated that Rydberg atoms in HCFs can be dressed by standard RF excitation methods, resulting in the occurrence of narrow spectral features for modulation frequencies up to 500 MHz^141^. More recently, the field has moved to using HCF in optical quantum memory applications. Such memories can store and retrieve quantum information carried by light on demand, which is crucial for developing quantum information science. Optical quantum memories using cold atoms as the storage medium exhibit high storage efficiency and long lifetimes. HCFs can provide the atomic ensemble with a quasi-monochromatic, non-diffractive light field, enabling strong light-atom interactions over long distances, making them an excellent medium for achieving quantum light storage and transmission^142–145^. Peters et al. reported on narrowband light storage and retrieval as well as stationary light for weak coherent light pulses down to the single-photon level based on electromagnetically induced transparency^144^. They conducted the experiments in an ensemble of laser-cooled atoms loaded into an HCF to provide strong light-matter coupling. Li et al. demonstrated a controlled transport of stored light using EIT in cold atoms within an HCF^143^. Light pulses are stored in an ensemble of cold rubidium atoms as dark-state polaritons (DSPs) and transported over 1.2 mm using an optical conveyor belt. They achieved a storage efficiency of up to 11% and a lifetime of 3.1 ms. The study highlights the robustness of light storage against dynamic acceleration and deceleration, suggesting further extensions to longer distances and more complex system.

### Distributed fiber sensing

**Fig. 10:**
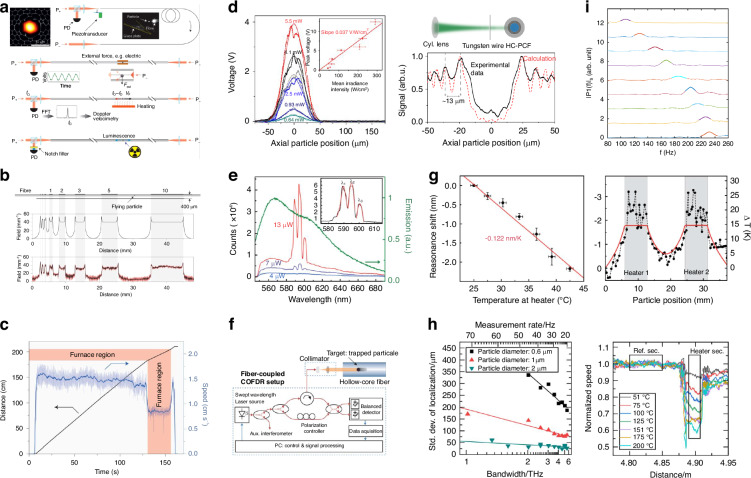
Distributed fiber sensors in HCF. **a** Sketch of flying particle sensors to detect electric field, temperature, and irradiation^71^. **b** Distributed electric field sensing using HCF-OT^71^. **c** Distributed temperature sensing using HCF-OT. The propelled particle slows down as it passes through the heated region^71^. **d** Distributed irradiation sensing using optically guided dye-doped microparticles in water-filled HCF^80^. **e** Flying microlaser in HCF. Stimulated emission peaks of the microlaser pumped at its absorption wavelength^146^. **g** Resonant shift of the lasing wavelength versus temperature (left panel) and the demonstration of distributed temperature sensing (right panel)^146^. **f** Combination of the HCF-OT and COFDR setup to achieve accurate particle localization^147^. **h** Standard deviation of the particle localization versus the measurement rate of COFDR (left panel) and instantaneous velocity of the 2 μm diameter silica particles flying over heated regime measured using COFDR (right panel)^147^. **i** Measured frequency of periodic motion of the particle due to the intermodal beating under different particle moving speed^148^

The levitated particle can be viewed as a point sensor, the motion status can be modified by environmental factors, such as temperature, electric and magnetic fields, acoustic waves, irradiation, etc. Combined with the capability of long-distance optical propulsion, the flying particle in the hollow core can be used as a multifunctional remote sensor [Fig. 10(a)]. Based on this idea, Bykov et al. have demonstrated the function of electric field and temperature sensing^71^. In the experiment of electric field sensing, a charged silica particle with a 3 μm diameter was propelled along a HC-PBG. The Columb forces induced by the external electric field can modify the equilibrium position of the trapped particle in the fiber cross-section, introducing the variation of the fiber transmission. The function of distributed electric field sensing has been demonstrated through a calibration of the modulation amplitude of the fiber transmission concerning the external electric field amplitude (Fig. 10(b)). In the case of temperature sensing, due to the temperature dependence of the air viscosity, particles entering the high-temperature region are expected to have a slower moving speed due to the higher viscous drag force. This principle can be used to realize a distributed temperature sensor, as shown in Fig. 10(c). Zeltner et al. reported a distributed radiation sensor based on a fluorescent flying particle optically propelled in the core of a water-filled HCF^80^ (Fig. 10(d)). When the moving particle passes through the irradiated region (UV radiation in the measurement), the excited fluorescence in the particle can be collected by the fiber core and transmitted to the photodiode at the endface, enabling the measurement of the spatial resolution of the radiation (bottom-left panel of Fig. 10(d)). By placing a tungsten wire in front of the radiation source to create a diffraction pattern, the spatial resolution of the sensor was measured as ~13 μm (bottom-right panel of Fig. 10(d)). Zeltner et al. demonstrated a distributed temperature sensor using a dye-doped polystyrene particle with a 15.5 μm diameter^146^. In this experiment, the whispering-gallery mode of the particle can form a laser cavity. When the particle is pumped at the absorption wavelength of the dye, as long as the pump power is above the lasing threshold, lasing peaks from the particle can be detected at the fluorescent wavelength of the doped dye, creating a flying microlaser (Fig. 10(e)). Due to the linear relation between the lasing wavelength shift and the local temperature variation, the function of distributed temperature sensing can be demonstrated (Fig. 10(g)). The achieved temperature measurement resolution is ~2.7 K, with a millimeter-scale spatial resolution.

The spatial resolution of the sensor is determined by the particle localization accuracy. When Doppler velocimetry is used, the error of particle localization may accumulate over time given the measurement uncertainty of the particle speed. To resolve this issue, Koepl et al. demonstrated a distributed temperature sensor by combining with coherent optical frequency domain reflectometry (COFDR)^147^ (Fig. 10(f)). The homemade COFDR system can simultaneously measure moving particles’ position and velocity. The localization accuracy of the particle is inversely proportional to the sweep bandwidth, as plotted on the left panel of Fig. 10(h), which can reach ~25 μm for a sweep bandwidth of 6 THz and 2 μm-diameter silica particle. Kincaid et al. reported the technique on the distributed temperature measurement by monitoring the average velocity of particles traveling through HCF^148^. The average speed of the particle was estimated by measuring the frequency of periodic motion of the particle due to the intermodal beating between LP_01_ and LP_11_ core modes (Fig. 10(i)). The measured average velocity of particles is a function of temperature due to the temperature dependence of the air viscosity in the hollow core. The technique has been applied to probe temperature distribution in hydrogen burners. Most recently, YIG/YFeO_3_ (Y_3_Fe_5_O_12_/ YFeO_3_) microspheres have also been trapped in HCF, which potentially can be used as a remote magnetic field sensor^149^. By monitoring the variations in intensity and polarization of the probe laser, the rotation of particles induced by light torque and the rotation of the magnetization vector in the presence of an external static magnetic field can be observed. The system could potentially be used to investigate levitate magneto-optical mechanics^150^.

The combination of optical guidance of the levitated particles and the sensing function can overcome several limitations of the existing distributed fiber sensors. First, the spatial resolution of the sensor is determined by the diameter of the trapped particle and its localization resolution, which can reach a micrometer or even nanometer scale^80,147^. On the other hand, the sensing distance is given by the transmission loss of the HCF, which may reach kilometers long when using ultra-low loss HC-ARF^108^. The decoupling between the spatial resolution and the sensing distance can resolve the long-term compromise between the two parameters in distributed fiber sensors. Second, the system function is determined by the type of the particle. The sensor can sense an electric field when a charged particle is used. Using the same piece of fiber, when the particle is replaced with one that can respond to a magnetic field, it can be viewed as a magnetic field sensor. The system can be viewed as a reconfigurable remote sensor.

### Particle metrology

An optical tweezer is a promising tool for characterizing the physical and chemical properties of the levitated particle at the single-particle level. This has been used in the characterization of nanoparticles^151,152^, cells^153–155^, viruses^156^, aerosols^11,50,157^ and microplastics^158,159^. The configuration of single-beam optical tweezers, especially the use of the high-numerical aperture objective to achieve stable trapping, can be directly multiplexed to realize the high-resolution imaging of the particle or to collect the weak scattered light and the Raman spectrum. This has led to the establishment of the field of single-particle spectroscopy^160,161^.

**Fig. 11:**
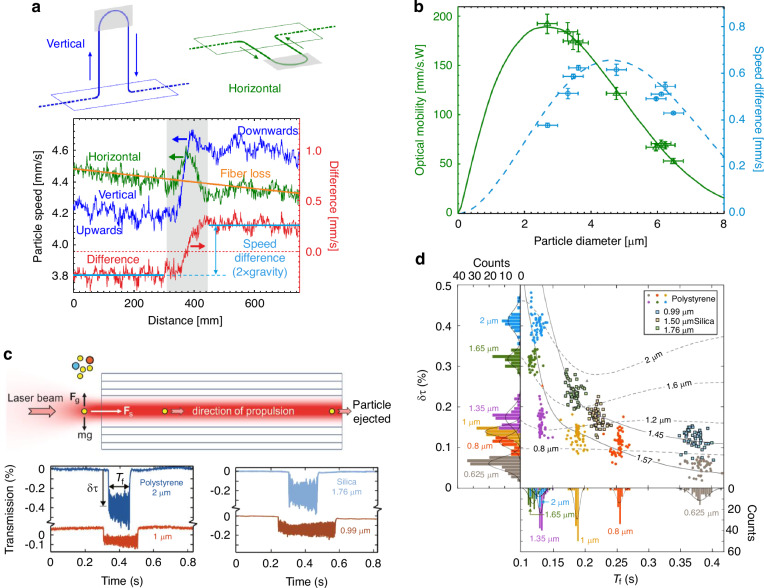
Single-particle characterization and metrologies using HCF-OT. **a** Vertical and horizontal arrangement of the fiber lengths used in the speed measurements (top panel). Particle speeds (left axis) and their difference (right axis) showing the effects of fiber loss and gravity (bottom panel)^125^. **b** Doppler-based measurements of optical mobility (triangles, left axis) and speed difference (circles, right axis) for different values of diameter. Comparison with theory (curves) allows determination of refractive index and mass density^125^. **c** Sketch illustrating the operating principle of the particle detector (top panel). Measured transmission drops δτ and time-of-flight *T*_f_ when polystyrene and silica particles of different diameter were detected^162^. **d** Two-dimensional scatterplot of δτ and *T*_f_ for polystyrene (dots) and silica (squares with black outlines) particles together with histograms, showing that particle different refractive index and diameter and well separated in the plot^162^

In HCF, the capability of long-distance particle propulsion adds a new degree of freedom to particle characterization. The precision balance of optical forces and the viscous drag forces can be used for a high-resolution retrieval of the particle properties, including the mass, diameter, and refractive index. Schmidt et al. developed a particle measurement technique based on the particle propulsion in HCF^125^. Under the condition of vertical and horizontal optical fiber with a bend section of 30 cm, the velocity difference of the particles in those two configurations was obtained by Doppler velocimetry. The diameter of the particles can be calculated from the optical mobility based on Stokes’ law and the equilibrium of the particles in the optical fiber when the particles are moving uniformly in the horizontal optical fiber. The difference in particle velocity between vertically and horizontally placed fibers can be calculated to determine the effect of gravity on particle velocity, and thus the particle density (Fig. 11(a), (b)). Sharma et al. reported a new technique offering in situ particle counting, sizing and refractive index measurement^162^. When an airborne particle enters the beam path, it is automatically captured by the gradient force in transverse direction and propelled through the fiber core by the scattering force generated from the LP_01_-like core mode. Once in the core, the particle scatters a fraction of the guided mode resulting in a drop in transmission that is detected by a photodiode, from which the amount of transmission drop and the time-of-flight can be resolved. Theory show that these two parameters can be unambiguously translated into particle diameter and refractive index with a high accuracy (Fig. 11(c), (d)). After detection, the particle simply ejects from the fiber without degrading the device. The technique can be directly applied to monitoring air pollution in the open atmosphere as well as precise particle characterization in a local environment.

### Biological particle manipulation

**Fig. 12:**
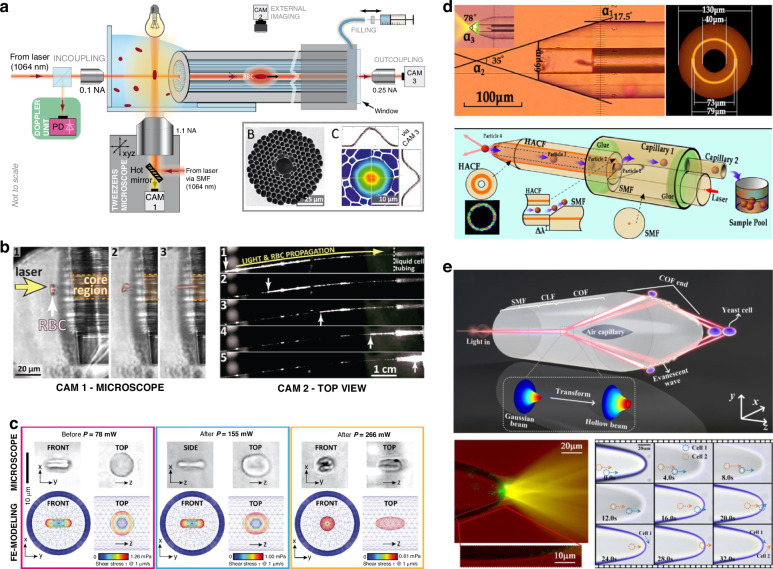
**Cell trapping and transporting in liquid-filled HCF**. **a** Optical propulsion of red blood cells in optofluidic HCF and cell speed monitoring by in-fiber Doppler velocimetry^163^. **b** Image results of red blood cells capture and propulsion^163^. **c** Experimental and finite element results of particle guidance under different optical powers^163^. **d** HCF-OT for cell manipulation and sterile transporting^95^. **e** HCF-OT with integrated ballistic transport and multi-point capture optical manipulation functions^165^

Filling liquid into the hollow core opens the possibility of realizing cell and virus trapping and probing the biological particles (cell, virus, etc.) in a well-confined environment. In 2012, Unterkofler et al. optically tweezered red blood cells (RBCs) and launched them into a liquid-filled HCF, and optically propelled them over several centimeters (Fig. 12a, b)^163^. The velocity of the cells was monitored by fiber-based Doppler velocimetry and observed to significantly increase once the intrinsically flat RBCs folded due to a combination of optical and fluidic forces (Fig. 12c). Complete interfolding of RBCs has only been observed in systems where the capillary is much smaller than the cell itself^164^, the HCF technique therefore complements normal studies on pressure-driven transport and deformation of RBCs in Poiseuille-flow.

As the simplest form of HCF, capillary fibers have also been used as a functional waveguide for cell trapping and sorting. In practical applications, it is always necessary to select one or more target cells for transfer from one biological environment (e.g., in cell culture) to another (e.g., in a cell detection system), as shown in Fig. 12(d). Zhang et al. proposed and demonstrated fiber optic tweezers based on hollow annular-core fiber (HACF), composed of a large diameter air hole in the middle and a annular waveguide in the middle of the capillary wall^95^. The propagation mode guided in the annular waveguide can be focused by the tapered fiber end and form a highly focused annular beam, thus leading to the manipulation of particle. And the hollow capillary of HACF naturally becomes a microfluidic channel which can realize particle transport in the fluid environment when a micro-pump is applied to the hollow hole of HACF. Therefore, the manipulation and transport of particles are realized by adjusting and controlling the resultant force of light trapping force and liquid viscous resistance. In addition, during the experiment, micro-pump can also be used to exert positive and negative pressure on the hollow hole of HACF, changing the speed and direction of the liquid, and adjusting the force of the liquid on the particle. It is ideal for the handling and sterile transport of rare cells. This kind of capillary fiber with a hollow hole and annular waveguide provides technical support for particle manipulation and transport simultaneously. This constitutes a new development of single fiber capture and allows more practical applications in particle manipulation and transport.

Integrating different optical manipulation functions into a single fiber is still challenging in the field of fiber-based optical tweezers. Deng et al. proposes a capillary optical fiber (COF) tweezer, which integrated ballistic transport and multi-point capture of yeast cells into a single fiber (Fig. 12e)^165^. The working principle is that the coreless fiber (CLF) segment first expands the conducting fundamental mode of the single-mode fiber (SMF). Then, the conical air cavity further dispersed the fundamental mode into a hollow beam composed of a series of higher-order guided modes. Then, at the interface between the envelope and the aqueous solution at the semi-ellipsoidal fiber end, the hollow beam is fully reflected, and the evanescent field generated will guide the yeast around the fiber end to transport along the surface to the fiber end. Finally, the hollow core beams converging through the semi-elliptical fiber end form a continuous multi-potential well region near the fiber end for stable yeast capture (Fig. 12e). This design enables the ballistic transport of multi-yeast cells along the surface of the cone tip, which are then trapped in multiple optical potential wells formed by the focused output beam. The integration of COF greatly enhances the function of fiber-based tweezer devices and provides a new tool for cell biology and medical research.

### Inertial sensing

**Fig. 13:**
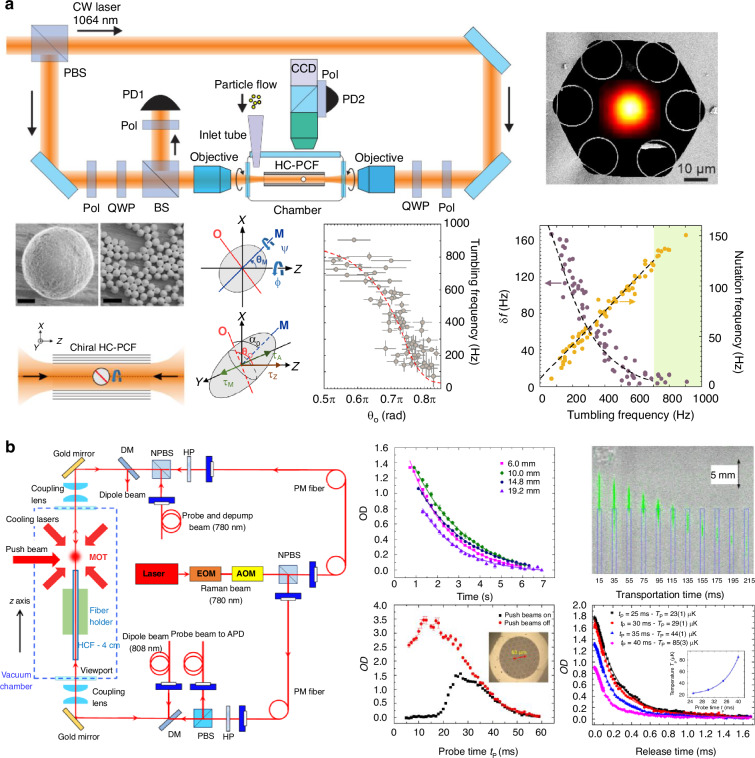
Rotational dynamics and inertial sensing using HCF-OT. **a** The observed tumbling, precession, and notation motions of vaterite microparticles in twisted HC-ARF^6^. **b** An inertia-sensitive atom interferometer capable of coherently guiding the ground-state superpositions of Rb atoms over centimeter distance and hundreds of milliseconds duration inside HCF^70,131^ (reprinted with permission from American Physical Society and AAAS)

The possibility of manipulating not only the translational but also the rotational motion of the objects can be used to investigate rotational dynamics and to realize a levitated micro-gyroscope. In a vacuum optical tweezer system, the rotation frequency of the levitated nanoparticle can already reach GHz level^166–168^. The rotational motion of the particle has shown the ability to stabilize the translational motions of the particles^9^. Single-beam optical tweezers have also been exploited to investigate the rich rotational dynamics of the levitated anisotropic nanoparticles^169–171^. In HCF, the protection of the core wall can isolate environmental perturbation and airflow well. Moreover, the fiber provides an ideal platform to develop a compact and fiber-integrated gyroscope. Sharma et al. reported the stable trap of birefringent vaterite particles in twisted HC-ARF^6^, in which the anti-resonant structure of the fiber is helically twisted along the fiber axis to maintain the spin angular momentum of the trapping beam. In this case, the circularly polarized light causes the anisotropic particles to spin about the fiber axis, while dipole forces tend to align the extraordinary optical axis of the vaterite particle into the plane of a rotating electric field. The result is that, accompanied by oscillatory nutation, the optical axis reaches a stable tilt angle with respect to the plane of the electric field. The tumbling, precession, and notation motions of the vaterite particles can be observed by monitoring the intensity and polarization modulation from the side or end face of the fiber. Such an optically levitated micromotor in HCF may be further used to achieve torque or orientation sensing (Fig. 13a). In the case of atom trapping, fiber-guided atom interferometers have shown excellent prospects in high-precision inertial sensing of gravity, gravity gradient, and acceleration^70,93,131,172,173^ (Fig. 13b). Compared with light-pulse atom interferometers in free space, fiber guided atom interferometers overcome the diffraction nature of light and have much stronger atom-photon interaction due to the tightly confinement of the atoms and photons inside the fiber. Xin et al. demonstrated an inertial-sensitive atom interferometer inside a hollow-core photonic crystal fiber in which the atomic wave-packets were spatially split, reflected and recombined^70^. The coherent time was limited to tens of micro seconds due to the inhomogeneous dephasing caused by the differential light shifts from the trapping beam on two interferometer arms. The inhomogeneous dephasing can be suppressed by further laser-cooling of the atoms inside the fiber or by applying another compensation laser beam that is mode matched to the trapping beam^93,173^. Moreover, since the fiber-guided atom interferometer measures the projection of gravity in the axial direction of the HCF, it is theoretically practical for the measurement of acceleration in arbitrary directions if the system itself can be rotated, and a vector fiber-guided atom gravimeter can be expected.

## Conclusion and Outlook

HCF represents a new platform progressing very fast to promote both the fundamental and application aspects of optical tweezers. The unique properties of HCF, especially the ultra-low transmission loss combined with the ability to manipulate the mode and optical force properties in the hollow core, offer opportunities for tailoring the long-range optomechanical interactions. Looking forward, HCF-OT still faces some challenges toward its wider applications.

### Tailoring the optical forces using HOMs in the hollow core

Fundamental core mode has been mainly used till now to achieve particle or atom trapping and measurement. Even though HOMs can be excited due to the imperfect coupling to the core, a controlled manner to excite the HOMs is expected to unlock new possibilities in the optomechanical control of the object motion. It has been shown that the excitation of HOMs with a reasonably high efficiency is possible for both air- and liquid-filled HCF through using a spatial light modulator^109,110^. Donut-shaped core modes may trap highly absorptive or metallic particles in HCF similar to that demonstrated in free-space optical tweezers. The multiple lobes of the HOMs may also be exploited to trap a 3D particle array in the fiber core.

### Tuning multiple-particle interactions in HCF

Optical trapping of an array of particles has drawn intense research interest in recent years due to the accessible non-Hermitian interactions that determine the collective dynamics of the particle array^174,175^. The formation of the particle array in HCF relies on the optical binding forces between the particles, in which the distances between particles are relatively large for the observation of Coulomb or dipole forces. One challenge is to develop a technique to precisely tune the distance and, thus, the interaction forces between the particles over a vast range in a controlled manner. This may greatly extend the number of particles and the types of forces (e.g. dipole force in the short range and Columb forces in the long range) involved in the investigation of the levitated collected optomechanics. Larger arrays of tunable interacting optically levitated particles have a great potential for studies of non-reciprocal phase transitions and non-equilibrium physics^175^.

### Hybrid optomechanics in HCF

Another interesting direction is to investigate the hybrid trapping of nanoparticles and atoms in the hollow core. The atom-nanoparticle system is expected to extend the demonstration of the quantum superposition principle to regimes of mass orders of magnitude larger than the current record^176^. Such a configuration has been reported in cavity optomechanical system, in which the quantum entanglement between the atom transition and the macroscopic particle motion could be observed^177^. The capability of guiding both atom clouds and nanoparticles in HCF, as summarized in this review, provides an ideal platform to explore short- and long-range entanglement features. A prerequisite to observing the phenomenon is the demonstration of the quantum ground state cooling of the levitated particles in HCF, such that sympathetic cooling of such a hybrid optomechanical system may be achieved.

In addition to the directions for the fundamental research aspect, several technical issues are essential for the future applications of HCF-OT:

### Sufficient loading of particles and atoms into HCF

The loading efficiency of the particle or atoms into the hollow core determines the successful rate of the optical trapping. Even though conditions for stable capture of the particle or atoms in front of the fiber endface have been revealed, there is still plenty of room for further boosting the loading efficiencies through, for instance, the combination of the optical forces and the hydrodynamics forces. Such a technique may greatly increase the feasibility of applying the HCF-OT technique on particle metrology, distributed fiber sensing and inertial sensing.

### Interfacing HCF with the high-vacuum environment

The ultra-high aspect ratio of the hollow core makes it challenging to remove residual gas molecules and create high vacuum level over the fiber core. A pressure gradient is expected to form given the boundary conditions of the pressure levels at the fiber ends^178,179^, while it may take minutes or even hours to reach equilibrium depending on the fiber length and core diameter. A proper technique to fasten the process would be highly beneficial for experiments of levitated optomechanics and atom trapping. In addition, techniques to provide a sealed optical tweezer device using integrated laser source and coupling optics are also in high demand for many applications, such as electric field sensing, and inertial sensing, to name a few.

### Recovery of particles attached to the core wall

Finally, developing the techniques to recover or remove the lost particle attached to the core wall would be highly useful for the practical application of HCF-OT technique. Even though the lost particle itself barely influences the propagation of the core mode, due to the very small overlap between the lost particle and the mode profile, the particle condemnation may influence the achievable vacuum level (depending on the material and number of the lost particles) and increase the loss rate of the subsequent particles. Techniques of launching hydrodynamic gas or liquid flows may be used to refresh the hollow core and to increase the lifetime of the HCF device.

## Data Availability

All data in the article are freely available upon request.
